# Posterior Teflon-Felt-Reinforced Coronary Button Anastomosis in a Modified Bentall Procedure: Early Outcomes in a Single-Center Retrospective Study

**DOI:** 10.3390/jcm15072546

**Published:** 2026-03-26

**Authors:** Özgür Akkaya, Izatullah Jalalzai, Ümit Arslan

**Affiliations:** 1 Department of Cardiovascular Surgery, Faculty of Medicine, Alanya Alaaddin Keykubat University, Antalya 07450, Türkiye; oakkaya369@gmail.com; 2 Department of Cardiovascular Surgery, Faculty of Medicine, Atatürk University, Erzurum 25030, Türkiye; ijalalzai@gmail.com

**Keywords:** modified Bentall procedure, aortic root surgery, coronary button, Teflon felt, hemostasis, bleeding, coronary reimplantation

## Abstract

**Background**: Coronary button reimplantation is a key determinant of operative safety in the modified Bentall procedure (MBP), and technical modifications aimed at improving anastomotic stability and hemostasis continue to evolve. This study investigated the early outcomes of a posterior Teflon-felt-reinforced coronary button technique in comparison with the conventional approach. **Methods**: Between January 2021 and May 2025, a total of 57 patients who underwent an elective modified Bentall procedure were included and divided into two groups: the conventional coronary button group (CCB, *n* = 30) and the posterior Teflon-felt-reinforced coronary button group (RCB, *n* = 27). Operative variables and early postoperative outcomes (including bleeding, re-exploration, and 30-day mortality) were compared between the two groups. **Results**: The CCB group included 9 women and 21 men with a mean age of 59.5 ± 9.6 years, whereas the RCB group consisted of 5 women and 22 men with a mean age of 57.3 ± 8.9 years. The mean maximum aortic root diameter was 49.6 ± 5.3 mm, and the mean ascending aortic diameter was 50.8 ± 4.9 mm. Aortic cross-clamp (ACC) and cardiopulmonary bypass (CPB) times were similar between the groups (*p* = 0.330 and *p* = 0.214, respectively). After excluding patients who underwent planned coronary artery bypass grafting (CABG; *n* = 8), the incidence of unplanned CABG was higher in the CCB group than in the RCB group [6 (24.0%) vs. 2 (8.3%); *p* = 0.136]. Postoperative 24-h chest tube drainage tended to be lower (*p* = 0.060), and re-exploration for bleeding occurred less frequently (11.1% vs. 30.0%, *p* = 0.076), with no coronary button-related bleeding after reinforcement. The RCB group required significantly fewer transfused blood products, including red blood cells, fresh frozen plasma, and platelets (all *p* < 0.01). Intensive care unit stay was shorter in the reinforced group (*p* < 0.01), with a trend toward reduced hospital stay (*p* = 0.085). Early mortality was comparable (*p* = 0.356). **Conclusions**: Posterior Teflon-felt-reinforced coronary button anastomosis may improve early hemostatic stability and provide additional mechanical support during coronary reimplantation in the modified Bentall procedure; confirmation in larger cohorts is required.

## 1. Introduction

In pathologies involving the aortic valve, aortic root, and ascending aorta, composite graft replacement with coronary artery reimplantation—originally described by Bentall and De Bono and subsequently refined by Cabrol, Kouchoukos, and others—has evolved into a durable cornerstone of contemporary aortic root surgery [1,2]. In patients for whom valve preservation is not feasible, the primary surgical objectives are to prevent operative mortality and to achieve reliable intraoperative hemostasis while minimizing perioperative morbidity [3].

Despite its long-established status as the standard approach, coronary button mobilization and reimplantation remain technically demanding, and ongoing refinements have largely focused on enhancing anastomotic integrity and long-term procedural durability [4,5,6]. Calcaterra et al. [7] demonstrated that prefabricated grafts with preformed coronary side branches can simplify coronary reimplantation, reduce the need for extensive mobilization, and facilitate the anastomosis, while also improving surgical exposure for hemostatic revision in fragile or dissected coronary ostia. However, because these grafts are not universally available, many centers continue to implement reproducible, surgeon-tailored technical modifications based on local resources and intraoperative requirements [8,9,10].

The success of aortic root replacement is largely determined by the quality of coronary button reimplantation, as excessive tension may compromise coronary perfusion, and anastomotic bleeding remains one of the most challenging intraoperative problems [11,12]. Achieving consistent and secure hemostasis at the coronary anastomosis is technically demanding, primarily because of the deep operative field and limited surgical exposure. To address these technical challenges, we adopted a modified Bentall approach incorporating a posterior Teflon-felt-reinforced coronary button anastomosis. The aim of this study was to evaluate its intraoperative hemostatic performance and to investigate the early postoperative clinical outcomes in comparison with those of the conventional button technique.

## 2. Materials and Methods

**Figure 1:**
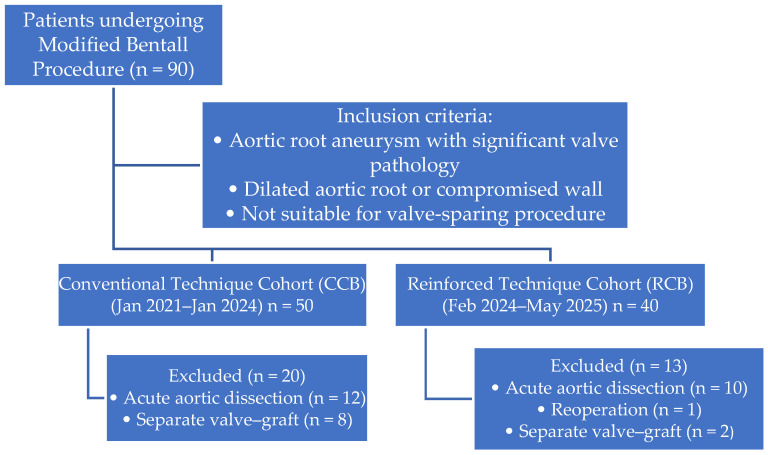
Flow diagram illustrating patient selection, inclusion and exclusion criteria.

This study included patients who underwent elective aortic root replacement with the modified Bentall procedure (MBP) for aortic root aneurysm between January 2021 and May 2025. The MBP was performed in patients with aortic root aneurysm associated with significant aortic valve pathology (including bicuspid aortic valve and severe aortic regurgitation), dilatation of the aortic root, or a structurally compromised aortic wall, in whom valve-sparing root replacement was not considered appropriate [13]. All operations were performed by the same senior surgeon (Ö.A.), ensuring technical consistency. The choice of coronary button technique (CCB vs. RCB) was not based on patient-specific clinical characteristics but reflected the temporal evolution of the surgical approach, with the reinforced technique adopted in the later period of the study. Among the 50 patients treated between 2021 and 2024, those operated on for acute aortic dissection (AAD; *n* = 12) and those who underwent separate graft configurations or valve-sparing procedures (*n* = 8) were excluded. The remaining 30 patients who underwent conventional coronary button reimplantation constituted the conventional technique group. Between 2024 and May 2025, 40 patients underwent MBP with posterior Teflon-felt-reinforced coronary button anastomosis. Of these, patients operated on for AAD (*n* = 10), those undergoing reoperative surgery (*n* = 1), and those who received a separate valve–graft configuration (*n* = 2) were excluded. The final reinforced cohort consisted of 27 consecutive patients who were prospectively enrolled. The study was designed to evaluate early postoperative outcomes, and data from both groups were compared accordingly. Patient selection and group classification are summarized in Figure 1.

Data were obtained from the institutional cardiovascular surgery database, in which preoperative, intraoperative, and postoperative variables are prospectively recorded. These data were supplemented by the operating surgeon’s prospectively maintained standardized logbook, which includes detailed operative reports and schematic surgical documentation for each procedure.

The Teflon-felt-reinforced coronary button (RCB) group was compared with the conventional coronary button (CCB) group with respect to baseline demographic characteristics, operative variables, and early postoperative outcomes. All patients underwent preoperative contrast-enhanced computed tomography for detailed evaluation of the aortic root and ascending aorta. In addition, coronary angiography was performed in patients older than 50 years, in those with at least two cardiovascular risk factors, or when coronary artery disease was suspected based on computed tomography findings. Aortic measurements were performed using a dedicated DICOM workstation (RadiAnt DICOM Viewer, Medixant, Poznań, Poland) and were independently assessed by an experienced cardiovascular radiologist and cardiovascular surgeons with at least 15 years of operative experience. The aortic root diameter was defined as the maximum diameter measured at the widest portion of the aortic root on contrast-enhanced computed tomography, regardless of the anatomical level. Operative data included aortic cross-clamp (ACC) time and cardiopulmonary bypass (CPB) duration. Postoperative outcomes comprised the total amount of chest tube drainage, the need for surgical re-exploration for bleeding, length of intensive care unit stay (days) and hospital stay (days), and early mortality. Postoperative bleeding was defined as chest tube drainage exceeding 1.5 mL/kg/h for 6 consecutive hours within the first 24 postoperative hours [14,15], and early mortality was defined as death occurring within 30 days postoperatively or during the index hospitalization.

### 2.1. Surgical Technique

**Figure 2:**
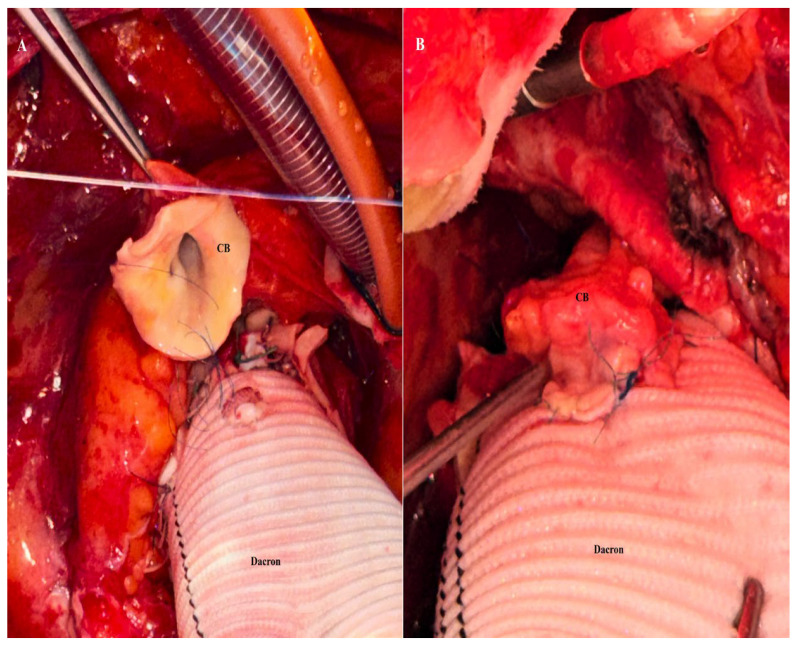
Conventional coronary button anastomosis to the Dacron conduit. (**A**) Conventional coronary button anastomosis to the Dacron conduit without additional buttressing, in which the native aortic cuff alone provides structural support and may be vulnerable to deformation or tearing under excessive traction. (**B**) Final appearance after completion of the conventional coronary button anastomosis to the Dacron graft using a double-suture technique, in which the anastomosis is reinforced by a second circumferential suture line and supported solely by the native aortic cuff without additional external buttressing. CB: coronary button; Dacron: vascular graft.

A standard median sternotomy was performed in all patients. Following systemic heparinization, arterial cannulation was established at the most cranial portion of the ascending aorta. Venous drainage was achieved using a two-stage venous cannula placed in the right atrium, and left ventricular venting was routinely performed through the right superior pulmonary vein. Cardiopulmonary bypass was conducted under moderate hypothermia. After aortic cross-clamping, cardiac arrest was achieved with cardioplegia administered either into the ascending aorta or selectively through the coronary ostia after aortotomy. The diseased aortic root and ascending aorta were resected, and the coronary ostia were excised as coronary buttons with an aortic cuff of approximately 5–8 mm. A composite graft—consisting of either a mechanical prosthetic valve mounted within a Dacron conduit (St. Jude Medical, Inc., St. Paul, MN, USA) or a biologic valve sutured to a woven Dacron graft and fashioned as a flanged composite conduit as previously described [16]—was implanted at the level of the left ventricular outflow tract. The coronary buttons were prepared to allow a tension-free anastomosis, and, after temporary cardiac filling, the optimal reimplantation sites on the graft were determined. In the CCB group, the coronary buttons were anastomosed to the graft in an end-to-side fashion using a continuous polypropylene suture [17] (Figure 2A,B).

**Figure 3:**
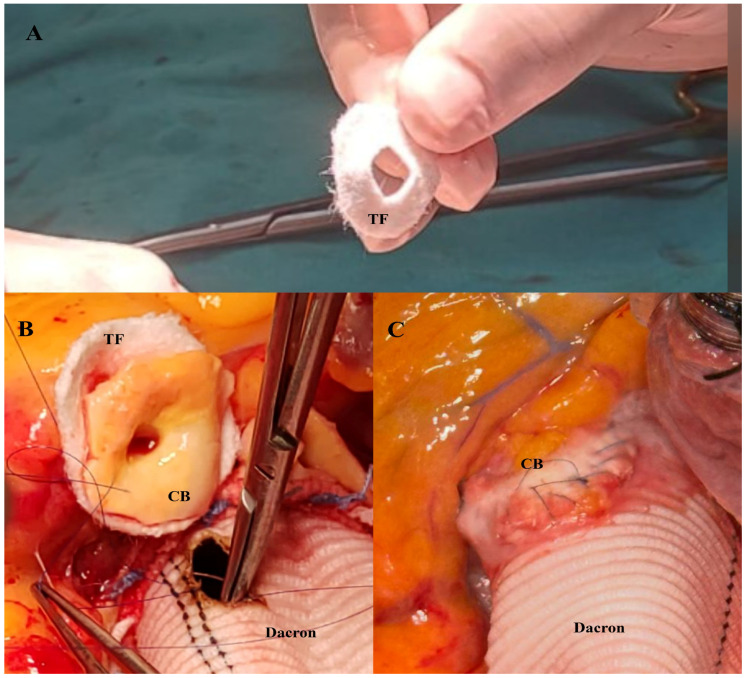
Preparation and application of posterior Teflon-felt reinforcement for the coronary button anastomosis. (**A**) Tailoring of the Teflon felt by creating a central opening sized to allow passage of the coronary button. (**B**) Positioning of the coronary button through the Teflon-felt ring, which is placed on the posterior aspect of the native aortic cuff to provide circumferential buttressing at the anastomotic interface. (**C**) Final appearance after completion of the coronary button anastomosis to the Dacron conduit with posterior Teflon-felt support. TF: Teflon felt; CB: coronary button; Dacron: vascular graft.

In the RCB group, a Teflon felt ring was tailored to match the size of the coronary button and positioned externally around the anastomotic site to provide circumferential support. The coronary button was then anastomosed to the graft together with the felt using a continuous 5-0 or 6-0 polypropylene suture, thereby creating a reinforced anastomotic line (Figure 3A–C). After completion of the coronary anastomoses, cardioplegia was delivered through the graft to assess anastomotic integrity and ensure meticulous hemostasis. The distal aortic anastomosis was subsequently constructed using a continuous 4-0 polypropylene suture.

Intraoperative assessment of coronary button integrity was performed using direct visual inspection and physiological parameters, including myocardial contractility, electrocardiographic findings, and the ability to achieve stable weaning from cardiopulmonary bypass. Particular attention was paid to the detection of technical issues such as kinking, torsion, or external compression of the coronary buttons. In the early postoperative period, patients were closely monitored for signs suggestive of coronary insufficiency or bleeding complications, including excessive chest tube drainage, cardiac tamponade, hemodynamic instability, and malignant ventricular arrhythmias. Patients with suspected complications were promptly taken for re-exploration. Routine postoperative imaging, including coronary computed tomography angiography or invasive coronary angiography, was not performed.

### 2.2. Statistical Analysis

Continuous variables were tested for normality using the Shapiro–Wilk test and are presented as mean ± standard deviation or median (Q1–Q3), as appropriate. Standardized mean differences (SMD) were calculated to quantify baseline differences between groups. Categorical variables are expressed as numbers and percentages. Between-group comparisons were performed using the independent-samples *t*-test with Welch correction for normally distributed variables and the Mann–Whitney U test for non-normally distributed variables. The chi-square test or Fisher’s exact test was used for categorical variables, as appropriate. Operative variables, postoperative bleeding parameters, transfusion requirements, and early clinical outcomes, including re-exploration for bleeding, length of intensive care unit and hospital stay, and 30-day mortality, were compared between the groups. Given the limited sample size, propensity score matching was not performed to avoid further reduction in statistical power and potential model overfitting. Instead, statistical methods appropriate for small-sample analyses were preferentially used. For binary outcomes with low event rates, Firth’s penalized likelihood logistic regression was applied to reduce small-sample bias and to obtain reliable effect estimates. A post hoc power analysis was conducted using G*Power software (version 3.1.9.7; Heinrich Heine University, Düsseldorf, Germany) based on the primary outcome measures (re-exploration for bleeding and unplanned coronary revascularization). The analysis demonstrated that the available sample size provided approximately 78% statistical power to detect moderate-to-large effect sizes between groups. All tests were two-sided, and a *p*-value < 0.05 was considered statistically significant. Statistical analyses were performed using Jamovi (version 2.7.13; The Jamovi Project, Sydney, Australia), with Firth models implemented in R (version 4.5.2, The R Foundation; Vienna, Austria).

## 3. Results

A total of 57 patients were included in the analysis, comprising 30 patients in the CCB group and 27 patients in the RCB group. The CCB group included 9 women and 21 men with a mean age of 59.5 ± 9.6 years, whereas the RCB group consisted of 5 women and 22 men with a mean age of 57.3 ± 8.9 years. There were no significant between-group differences in sex distribution (*p* = 0.315) or age (*p* = 0.390, Welch’s *t*-test), and the effect size for age was small (Cohen’s d = 0.23; 95% CI, −0.2 to 0.7).

The most common presenting symptoms were exertional fatigue, dyspnea, and chest pain. Four patients were diagnosed during evaluation for a marfanoid phenotype, and eight were referred for further cardiovascular assessment after failure to obtain an adequate arterial blood pressure measurement. Hypertension was present in 44 patients and diabetes mellitus in 11. Thirty patients were active smokers, 25 of whom were receiving medical therapy for chronic obstructive pulmonary disease.

Annulo-aortic ectasia was a frequent finding, with a mean maximum aortic root diameter of 49.6 ± 5.3 mm and a mean ascending aortic diameter of 50.8 ± 4.9 mm. Severe aortic regurgitation was present in the majority of patients and was associated with a larger mean aortic diameter (54.1 ± 3.6 mm), whereas patients with a bicuspid aortic valve (*n* = 18) had a smaller mean aortic diameter (47.6 ± 2.8 mm). Baseline demographic and clinical characteristics are summarized in Table 1.

All patients underwent surgery through a median sternotomy with arterial cannulation of the ascending aorta and venous cannulation of the right atrium. ACC time was comparable between the groups [120 (105–145) vs. 115 (105–122) min; *p* = 0.330; rank-biserial r = −0.185]. Similarly, CPB time did not differ significantly [180 (164–195) vs. 165 (148–193) min; *p* = 0.214; rank-biserial r = −0.235]. Concomitant planned coronary artery bypass grafting (CABG) for three-vessel coronary artery disease was performed in five patients in the CCB group and in three patients in the RCB group.

In the CCB group, six patients could not be weaned from CPB and therefore required additional intraoperative coronary revascularization. Saphenous vein grafting was performed to the left coronary system in two patients and to the mid–right coronary artery in four patients. In the RCB group, two patients underwent saphenous vein grafting to the right coronary artery to achieve successful separation from bypass. Intra-aortic balloon pump support was instituted in the two patients who required revascularization of the left coronary system. After exclusion of patients who underwent planned concomitant CABG, the incidence of unplanned intraoperative coronary revascularization was higher in the CCB group than in the RCB group [6 (24.0%) vs. 2 (8.3%); *p* = 0.136]. In Firth’s penalized logistic regression analysis, the reinforced coronary button technique was associated with a lower likelihood of additional coronary revascularization (OR 0.33, 95% CI 0.06–1.74; *p* = 0.191).

Hemostatic control of the proximal graft anastomosis was achieved in all patients using a double-pledgeted suture technique, while circular pericardial patch support was applied at the distal anastomosis. Intraoperative additional suturing at the coronary button anastomosis was required in nine patients in the conventional group. In the reinforced group, additional sutures were needed in two patients due to minor kinking and uneven configuration of the right coronary button. No coronary button required takedown and reimplantation in any patient.

During the first 24 postoperative hours, cumulative chest tube drainage tended to be lower in the RCB group than in the CCB group [380 (290–460) vs. 425 (350–650) mL; *p* = 0.060; rank-biserial r = −0.275]. In parallel, the RCB group required significantly fewer transfused blood products, including red blood cells [4 (2.5–5.0) vs. 2 (1.0–4.0) units], fresh frozen plasma [6 (5.0–9.0) vs. 4 (3.0–5.0) units], and platelets [2 (1.0–2.0) vs. 1 (0.0–1.0) units] (all *p* < 0.01). Re-exploration for bleeding was required in nine patients (30.0%) in the CCB group and in three patients (11.1%) in the RCB group (Fisher’s exact test, *p* = 0.076). In Firth’s penalized logistic regression analysis, the reinforced coronary button technique was associated with a lower likelihood of re-exploration (OR 0.34, 95% CI 0.08–1.36; *p* = 0.130). At re-exploration, no coronary button-related bleeding was observed in the RCB group; the identified sources were distal anastomotic bleeding in one patient and diffuse mediastinal bleeding in two patients. In contrast, coronary button bleeding occurred only in the CCB group (*n* = 2). Additional sources in this group included proximal suture line bleeding in two patients and diffuse mediastinal bleeding in five patients. Both patients with proximal suture line bleeding had undergone concomitant CABG.

Early mortality occurred in 5 patients (8.8%), three of whom were women. One death was observed in the RCB group and four in the CCB group (*p* =0.356). In Firth’s penalized logistic regression analysis, the reinforced coronary button technique was not significantly associated with early mortality (OR 0.33; 95% CI 0.03–1.96; *p* = 0.200).

In the RCB group, the deceased patient was a 75-year-old woman who underwent re-exploration for bleeding and died on postoperative day 3 due to consumptive coagulopathy. In the CCB group, a 68-year-old woman died on postoperative day 2 due to low cardiac output syndrome. A 77-year-old man who required intraoperative revascularization of the left coronary system and postoperative intra-aortic balloon pump support developed low cardiac output complicated by acute renal failure and died on postoperative day 3. Two additional in-hospital deaths occurred later in the postoperative course: a woman with concomitant coronary artery disease died on postoperative day 10, and an 81-year-old man died due to a cerebrovascular event.

The length of intensive care unit stay was significantly shorter in the RCB group than in the CCB group [3.0 (3.0–3.75) vs. 4.0 (4.0–5.0) days; *p* < 0.01]. Total hospital stay was also shorter in the RCB group, although the difference did not reach statistical significance [7.0 (7.0–7.75) vs. 8.0 (7.0–11.0) days; *p* = 0.085]. The intraoperative data and early postoperative outcomes of both groups are presented in Table 2.

## 4. Discussion

This study evaluated the impact of posterior Teflon-felt reinforcement of coronary buttons, excised with a 5–8 mm cuff of native aortic wall, on early clinical outcomes in patients undergoing the MBP. In this technique, the coronary buttons were supported posteriorly with a circular Teflon-felt pledget prior to reimplantation. Compared with the conventional button technique, the reinforced approach was associated with a lower incidence of unplanned intraoperative CABG (8.3% vs. 24.0%; OR 0.33, 95% CI 0.06–1.74; *p* = 0.191), a reduced rate of re-exploration for bleeding (11.1% vs. 30.0%; OR 0.34, 95% CI 0.08–1.36; *p* = 0.130), and a numerically lower early mortality (3.7% vs. 13.3%; OR 0.33, 95% CI 0.03–1.96; *p* = 0.200). In addition, the reinforced group had a significantly shorter intensive care unit stay and showed a trend toward lower postoperative chest tube drainage and shorter total hospital stay, without prolongation of operative times. These findings suggest that posterior Teflon-felt buttressing provides structural stabilization of the coronary button during reimplantation, thereby reducing the risk of intimal injury and minimizing the need for additional hemostatic or repair sutures when adequate coronary mobilization is achieved.

For conditions involving both the aortic valve and the aortic root—most notably annuloaortic ectasia and aortic dissection—composite valved conduit replacement has become the preferred surgical strategy [18]. Since its original description by Bentall and De Bono, the procedure has undergone substantial refinement, particularly with the modifications introduced by Kouchoukos and Karp, who standardized coronary reimplantation using the button or open anastomosis technique, which continues to define contemporary surgical practice [19,20].

Replacement of the aortic root entails reconstruction of the origin of the systemic circulation and is therefore inherently associated with considerable operative risk. In this context, coronary reimplantation constitutes a critical step, as it directly determines myocardial perfusion and the ability to achieve safe separation from CPB [21]. For nearly six decades since the original description of the Bentall procedure, the success of this operation has depended not only on conduit implantation but also on meticulous preparation and reattachment of the coronary buttons [22,23,24]. Accordingly, technical refinements—driven by cumulative surgical experience and progressive procedural optimization—have primarily focused on achieving reliable coronary reimplantation, minimizing anastomotic bleeding, and preventing coronary-related postoperative complications [17,25].

In the MBP, coronary reimplantation requires meticulous preparation of the coronary buttons, sufficient mobilization to avoid kinking or excessive tension, precise alignment with the crimped structure of the Dacron conduit, and atraumatic suturing of the fragile tissue in a manner that preserves luminal geometry, maintains ostial patency, and ensures reliable hemostasis [26,27]. In an analysis of more than 8000 elective aortic root operations, Wallen et al. [28] demonstrated that factors reflecting coronary compromise—most notably the need for additional CABG—were strongly associated with increased mortality. In aortic root aneurysm, the native course of the coronary arteries is distorted, and reimplantation requires their adaptation to a reconstructed systemic outflow tract; inaccurate positioning may result in tension, kinking, or torsion, thereby compromising coronary flow and myocardial perfusion. Furthermore, mechanical stress on the fragile coronary button may lead to intimal injury, tissue tearing, and anastomotic bleeding, frequently necessitating additional corrective procedures and adversely affecting early clinical outcomes [29,30,31]. In a contemporary series, Rajesh et al. [32] reported a rate of unplanned CABG of 8.4%, predominantly attributable to technical and anatomical challenges related to coronary button preparation and reimplantation, including friable tissue, ostial involvement, mobilization injury, and impaired coronary flow at the anastomotic site. These technical and anatomical challenges constitute the rationale for strategies aimed at reinforcing the coronary button and stabilizing the anastomotic interface.

Aortic pathologies may manifest with coronary ischemia due to altered coronary anatomy associated with aneurysmal disease or extension of aortic dissection into the coronary ostia. Furthermore, early postoperative coronary obstruction following aortic root surgery may result in acute myocardial ischemia, ventricular arrhythmias, myocardial infarction, and heart failure, potentially leading to life-threatening complications [33,34,35]. Saxena et al. [36] reported the use of percutaneous coronary stenting in a hemodynamically unstable patient with left main coronary artery occlusion to achieve initial stabilization, followed by definitive surgical intervention. Preoperative percutaneous coronary interventions may serve as an important bridging strategy by providing hemodynamic stabilization prior to definitive surgical management [37,38]. However, despite the potential role of preoperative coronary stenting, the importance and technical significance of coronary reimplantation in aortic root surgery remain paramount [39]. In cases of failed coronary perfusion following coronary reimplantation, clinical presentation may include inability to wean from cardiopulmonary bypass or the development of life-threatening ventricular arrhythmias after separation from bypass or during the early postoperative period. In such situations, percutaneous coronary intervention is often not feasible, and restoration of coronary blood flow requires urgent surgical revascularization using the internal mammary artery (IMA) or saphenous vein grafts (SVG) [40,41]. The use of the left internal mammary artery (LIMA) during cardiopulmonary bypass may be a reasonable option; however, in situations arising after decannulation, preparing a saphenous vein graft (SVG) through simultaneous harvesting during cannulation may offer a time-saving advantage. Ogami et al. [42] reported that saphenous vein grafts were utilized in 85.3% of unplanned coronary artery bypass procedures, a high proportion that appears to be largely attributable to the predominance of right coronary artery revascularization (76.5%). In aortic root procedures such as the modified Bentall operation, the addition of concomitant CABG has been associated with increased requirements for positive inotropic and mechanical circulatory support, as well as higher rates of morbidity and mortality [43]. However, adequate myocardial protection, minimization of ischemic time, and timely and appropriate surgical strategy may improve the feasibility and safety of CABG in this setting [44].

In our aortic root surgery, coronary mobilization is performed in a controlled and balanced manner, sufficient to avoid both excessive traction and inadequate release while preserving collateral circulation. The Dacron conduit is tailored to an appropriate distal length, and the optimal sites for coronary reimplantation are determined with the heart filled to reproduce physiological geometry. After completion of the left coronary anastomosis, the graft is gently aligned longitudinally toward the distal aorta, and the heart is refilled to allow physiologically oriented and tension-free implantation of the right coronary artery [4,25]. In our initial experience, the coronary buttons were anastomosed with a generous cuff of native aortic wall. However, because a prosthetic graft lacks the biological and mechanical properties of living aortic tissue, the anastomosis does not constitute a true tissue-to-tissue interface, which may limit the formation of a uniformly supported and hemostatically reliable suture line.

Since 2024, we have adopted posterior Teflon-felt reinforcement of the coronary button to provide a stable biological buttress at the anastomotic site, thereby enhancing structural support and largely eliminating the need for additional hemostatic sutures without prolonging ACC or CPB times. Although the number of patients was limited, additional right coronary saphenous vein grafting was required in only two cases in the reinforced group (8.3%, *n* = 2/24). This most likely reflects a protective strategy in the presence of intraoperative concern regarding coronary flow and the well-recognized low threshold for right coronary revascularization to facilitate safe separation from CPB, rather than failure of the reinforced anastomosis [42].

Bleeding represents another major challenge in aortic root surgery, because once the aortic cross-clamp has been removed and full myocardial reperfusion is established, precise localization and secure control of hemorrhagic sites—particularly at the coronary button anastomoses—become considerably more demanding [45]. Mookhoek et al. [46] reported a re-exploration rate for bleeding of 6.7%, with values in the literature ranging from 0% to 23%, underscoring the heterogeneity of surgical techniques and the persistent difficulty in achieving consistent perioperative hemostasis in aortic root procedures. Beyond its systemic consequences, bleeding from the coronary button frequently necessitates additional hemostatic sutures or the application of topical sealants. These measures may alter the spatial orientation and geometry of the reimplanted coronary artery, lead to external compression or deformation, and—through local inflammatory reactions to biological adhesives—further compromise coronary perfusion [47,48,49].

Adhesive materials may induce local tissue reactions and exhibit variable adherence properties influenced by hemodynamic conditions; when used excessively or aggressively, they may exert external compression on the coronary arteries or lead to embolic complications, thereby compromising coronary perfusion [50,51]. In particular, ostial stenosis, pseudoaneurysm formation, or torsion of the left coronary system may result in life-threatening complications and necessitate reintervention [48,52]. Therefore, Anastasius et al. [53] have advised against the use of surgical adhesives in this setting. The use of polytetrafluoroethylene (PTFE)-based Teflon felt has become widespread to reinforce fragile tissues and to prevent blood leakage through the interstices of polypropylene sutures [54]. In an experimental study by No et al. [55], Teflon felt reinforcement was shown to enhance the tensile strength and mechanical stability of anastomotic constructs, thereby improving resistance to suture line failure. However, reoperative aortic root surgery has become increasingly common over time, and coronary dissection in the presence of dense adhesions represents one of the most critical technical challenges [56]. This difficulty may reflect the effects of surgical adhesives, as Head et al. [57], in a meta-analysis, reported that Teflon may be effective in preventing adhesion formation. No reoperative cases were observed in our series following the use of the posterior Teflon-felt-reinforced coronary button technique. However, circumferential posterior reinforcement of the coronary buttons may facilitate coronary exposure in potential redo operations and may allow easier application of the coronary button preparation technique described by Ohira et al. [58].

Alternative technical strategies should be considered in the presence of concerns regarding the structural integrity of the coronary artery–graft anastomosis or in situations associated with a high risk of clinically significant suture line bleeding [17,59]. Li et al. [60] described a complex reconstructive strategy in which the coronary buttons were reimplanted into a valved conduit, the aortic sinuses were reconstructed with bovine pericardium, and the residual aortic wall was wrapped to create an internal shunt to the right atrium. Although this approach may be advantageous in acute aortic dissection with extremely fragile tissues, its technical complexity may limit its routine applicability. In a subsequent modification, Li et al. [61] anastomosed the coronary buttons to circular Dacron patches tailored from the valved conduit and then implanted these composite patch–coronary units into the graft, again combined with wrapping of the native aortic wall to facilitate hemostatic control.

In contrast, our technique provides posterior buttressing of the coronary button using a Teflon-felt ring tailored to the ostial size and sutured together with the button to the Dacron conduit after complete excision of the diseased aortic tissue, without wrapping of the residual aortic wall. No coronary button-related bleeding was observed in the reinforced group, whereas two patients in the conventional group required re-exploration for bleeding originating from the right coronary button. In Firth’s penalized logistic regression analysis, the reinforced technique was associated with a lower likelihood of bleeding-related re-exploration (OR 0.34, 95% CI 0.08–1.36; *p* = 0.130), although this did not reach statistical significance.

In addition to surgical technique, several factors may influence postoperative bleeding in aortic surgery, including advanced age, low preoperative hemoglobin levels, coagulation disorders, platelet dysfunction, and systemic inflammatory responses [62]. Prolonged cardiopulmonary bypass-related hypothermia not only impairs endothelial function but may also activate cold-reactive proteins that interact with surface antigens on erythrocytes, B and T lymphocytes, monocytes, and neutrophils [63]. These include cryoglobulins, cold agglutinins, Donath–Landsteiner antibodies, and cryofibrinogen. Upon exposure to low temperatures, these factors may induce hemagglutination and complement activation, followed by hemolysis particularly during the rewarming phase. Although relatively uncommon, such mechanisms have been reported to contribute to perioperative bleeding complications in a small subset of patients, with an estimated incidence ranging from approximately 1% to 4% [64,65]. Individualized preoperative planning, minimizing the duration of hypothermia, the use of warm blood cardioplegia, and, in selected cases, targeted therapies such as humanized monoclonal antibodies may help reduce the risk of bleeding in cardiac surgery [66,67].

As the MBP entails reconstruction of the ascending aorta, aortic valve replacement, and coronary reimplantation, it is inherently associated with prolonged cardiopulmonary bypass and extensive tissue manipulation, both of which may contribute to an increased risk of bleeding. This tendency may be further amplified by transfusion requirements and coagulation disturbances, ultimately leading to a vicious cycle of bleeding. In this context, mediastinal packing combined with delayed sternal closure (DSC) represents an established and widely adopted strategy for the management of refractory perioperative bleeding following complex cardiac and proximal aortic surgery, providing effective hemostatic control while allowing physiological stabilization prior to definitive closure [68]. In the study by Iscan et al. [69], delayed sternal closure was performed in 124 patients, with bleeding representing the primary indication in 65% of cases. These patients were characterized by longer aortic cross-clamp and cardiopulmonary bypass durations and a greater requirement for hemodynamic support. Although sternal management strategies are effective in emergency settings such as aortic dissection and in other technically demanding procedures, maintaining sternal stability and preventing sternal wound complications remain critical considerations [70,71]. Furthermore, in patients requiring cardiopulmonary resuscitation or urgent re-exploration following cardiac surgery, comprehensive chest wall stabilization may be necessary to prevent sternal complications, which can otherwise lead to respiratory dysfunction, infectious sequelae, and even mortality [72,73]. All patients included in the present study underwent elective surgery, and delayed sternal closure was not required in any case. In patients who required re-exploration, chest wall stabilization was performed concomitantly. Depending on the clinical condition, stabilization was achieved using titanium plate fixation or polymer cable systems, and pectoralis muscle flap reconstruction was employed when deemed necessary [74].

Given the inherent complexity of the MBP, meticulous coronary handling, a stepwise and patient-specific operative strategy, and restoration of a physiological coronary course are essential for procedural safety [75,76]. In our approach, the coronary ostia are excised as buttons with an approximately 5–8 mm cuff of native aortic tissue and anastomosed to the Dacron conduit using a tailored posterior Teflon-felt ring that functions as a buttress at the anastomotic interface. This configuration creates a uniformly supported suture line, enhances hemostatic stability at the time of the initial implantation, and preserves the spatial orientation of the reimplanted coronary arteries. By providing primary structural support, the technique reduces the need for secondary hemostatic maneuvers that may otherwise distort coronary geometry or compromise myocardial perfusion [77]. In this regard, posterior Teflon-felt buttressing represents a practical technical refinement that directly addresses one of the most demanding steps of aortic root replacement.

## 5. Limitations

The present study should be interpreted in the context of several limitations. The relatively small sample size, resulting from the deliberate inclusion of a homogeneous cohort of patients undergoing the modified Bentall procedure for aortic root aneurysm with significant aortic regurgitation and the exclusion of acute aortic dissection to minimize heterogeneity in inflammatory status and bleeding tendency, may have reduced statistical power and introduced a degree of selection bias. Although a post hoc power analysis indicated that the study had sufficient power to detect moderate-to-large effect sizes, it may have been underpowered to identify smaller between-group differences, which could explain the borderline statistical significance observed in some outcomes. All procedures were performed by a single experienced surgeon, ensuring technical consistency but potentially limiting the generalizability of the findings across different surgical teams and institutional settings [78]. In addition, the single-center design represents a further limitation. Finally, the analysis was restricted to early postoperative outcomes due to the relatively small sample size and limited follow-up duration; therefore, the impact of this technique on long-term coronary patency, anastomotic durability, and late clinical outcomes remains to be determined in larger studies with extended follow-up.

## 6. Conclusions

Posterior reinforcement of the coronary button with a tailored Teflon-felt ring was associated with reduced bleeding-related events compared with the conventional button technique, without prolonging aortic cross-clamp or cardiopulmonary bypass times and with only a limited need for unplanned right coronary bypass. No coronary button-related bleeding was observed in the reinforced group. This modification was accompanied by shorter intensive care unit and hospital stay, suggesting a potential improvement in early postoperative recovery. These findings indicate that this simple and reproducible technique may provide additional mechanical support at the coronary anastomosis, particularly in the presence of fragile aortic tissue, while minimizing the need for supplementary hemostatic sutures and excessive coronary manipulation. Larger studies with longer follow-up are required to confirm its impact on clinical outcomes.

## Figures and Tables

**Table 1:** Preoperative baseline characteristics of the study groups.

Variable	CCB Group (*n* = 30)	RCB Group (*n* = 27)	*p*
Age (years)	59.5 ± 9.6	57.3 ± 8.9	0.390
Female sex, *n* (%)	9 (30.0)	5 (18.5)	0.315
BMI (kg/m^2^)	26.8 ± 3.1	27.1 ± 2.7	0.875
Hypertension, *n* (%)	21 (70.0)	23 (85.2)	0.147
Diabetes mellitus, *n* (%)	6 (20.0)	5 (18.5)	0.578
Current smoker, *n* (%)	17 (56.7)	13 (48.1)	0.112
COPD, *n* (%)	15 (50.0)	10 (37.0)	0.352
Coronary artery disease, *n* (%)	5 (16.7)	3 (11.1)	0.415
Bicuspid aortic valve, *n* (%)	10 (33.3)	8 (29.6)	0.784
LVEF (%)	49.5 ± 5.4	49.0 ± 6.5	0.619
EuroSCORE II (%)	2.5 ± 1.6	2.8 ± 2.2	0.548
Maximum aortic root diameter (mm)	49.9 ± 5.6	49.2 ± 5.0	0.620
Ascending aorta diameter (mm)	51.1 ± 5.1	50.4 ± 4.7	0.591

BMI: Body Mass Index; COPD, chronic obstructive pulmonary disease; LVEF, left ventricular ejection fraction; CCB, conventional coronary button group; RCB, reinforced coronary button group.

**Table 2:** Intraoperative data and early postoperative outcomes.

	CCB Group (*n* = 30)	RCB Group (*n* = 27)	*p*
Intraoperative Data			
Aortic cross-clamp time (min) ^a^	120 (105–145)	115 (105–122)	0.330
Cardiopulmonary bypass time (min) ^a^	180 (164–195)	165 (148–193)	0.214
Planned CABG (three-vessel), *n* (%)	5 (16.7)	3 (11.1)	0.415
* Unplanned intraoperative CABG, *n* (%)	6 (24)	2 (8.3)	0.136
Intra-aortic balloon pump support, *n* (%)	2 (6.7)	0 (0)	0.153
Early postoperative outcomes			
Chest tube drainage, first 24 h (mL ^a^)	425 (350–650)	380 (290–460)	0.060
RBC transfused, units ^a^	4 (2.5–5.0)	2 (1.0–4.0)	<0.01
FFP transfused, units ^a^	6 (5.0–9.0)	4 (3.0–5.0)	<0.01
PLT transfused, units ^a^	2 (1.0–2.0)	1 (0.0–1.0)	<0.01
Re-exploration for bleeding, *n* (%)	9 (30.0)	3 (11.1)	0.076
Length of ICU stay (days) ^a^	4.0 (4.0–5.0)	3.0 (3.0–3.75)	<0.01
Length of hospital stay (days) ^a^	8.0 (7.0–11.0)	7.0 (7.0–7.75)	0.085
Early mortality, *n* (%)	4 (13.3)	1 (3.7)	0.356

CABG, coronary artery bypass grafting; FFP, fresh frozen plasma; ICU, intensive care unit; PLT, platelet; RBC, red blood cell. ^a^ Values are presented as median (Q1–Q3) * Percentages were calculated after exclusion of patients who underwent planned concomitant CABG.

## Data Availability

All data generated or analyzed during this study, including imaging materials, laboratory findings, and statistical data, are securely stored within the authors’ archives. No data were obtained from external sources or previously published materials. These datasets are available from the corresponding author upon reasonable request.

## Abbreviations

CCB	Conventional coronary button
RCB	Reinforced coronary button
MBP	Modified Bentall procedure
CPB	Cardiopulmonary bypass
ACC	Aortic cross-clamp
CABG	Coronary artery bypass grafting
